# Supplementary material for: Three-dimensional localization of nanoscale battery reactions using soft X-ray tomography
Source: Nat Commun. 2018 Mar 2;9:921. doi: 10.1038/s41467-018-03401-x (PMC5834601; doi:10.1038/s41467-018-03401-x)
Supplement: Supplementary file 1 — Supplementary Information [file 41467_2018_3401_MOESM1_ESM.pdf]

This PDF File includes:

Supplementary Movies 1

Supplementary Figs 1–14

Supplementary Method

Supplementary Reference

Supplementary Movie 1. Reconstructed 3-dimensional (3D) volumes of optical density (gray), chemical maps (red-blue), and its segmentation (red-green-blue). The size of reconstructed voxels is 6.7 × 6.7 × 6.7 nm^3. The 3D edge contrast of the optical density volume is enhanced for clear visibility. The presence of the $Li_{\alpha}FePO_4$ (majority Fe^{2+}, LFP) and charged $Li_{\beta}FePO_4$ (majority Fe^{3+}, FP) were assigned colors red and blue, respectively (chemical map). The red, green, and blue areas indicate LFP-rich (>70% $Li_{\alpha}FePO_4$), FP-rich (>70% $Li_{\beta}FePO_4$), and Mixed (30–70% $Li_{\alpha}FePO_4$, the rest being $Li_{\beta}FePO_4$) domains, respectively (segmented chemical map).

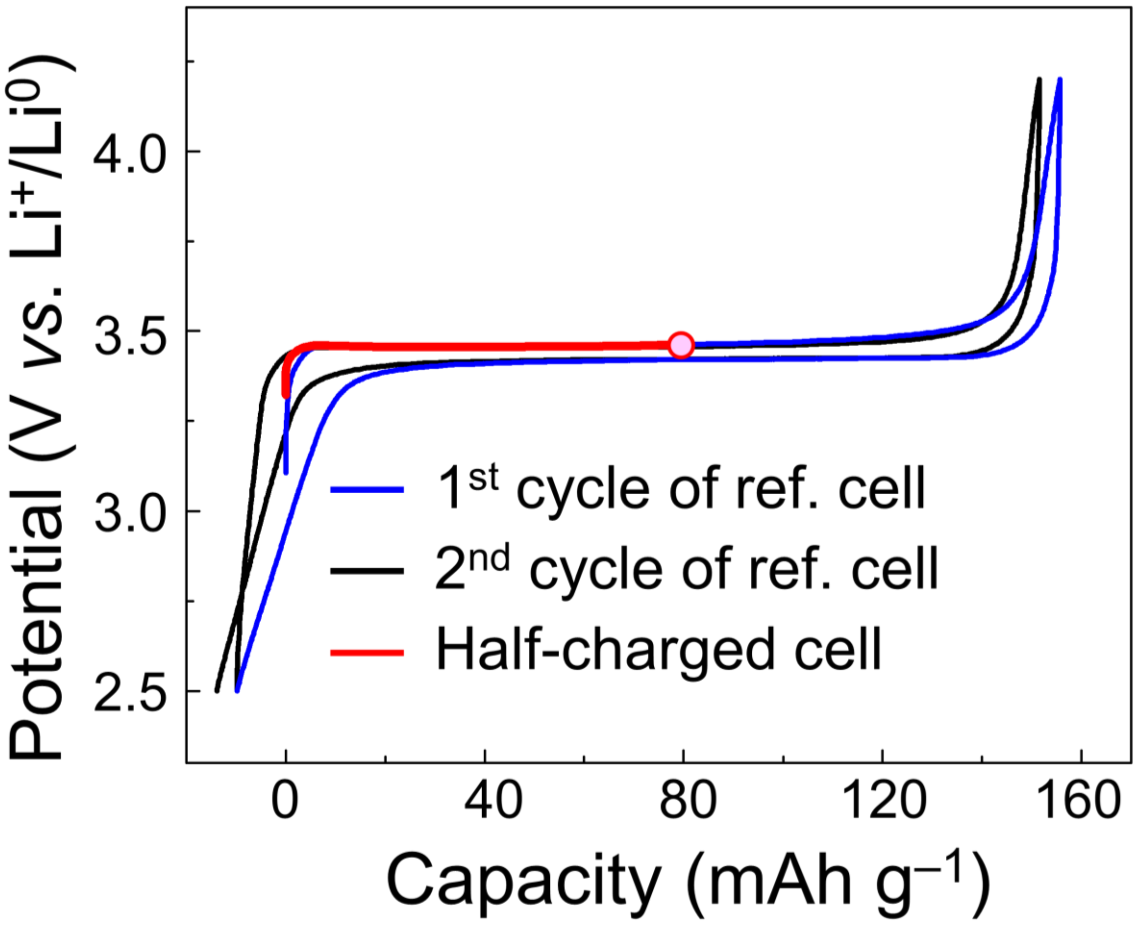

Supplementary Figure 1. Galvanostatic charge/discharge profiles of the first (blue solid line) and second (black solid line) cycles of $LiFePO_4$ nano-plates under normal cycling conditions (room temperature, C/10 rate). Li_xFePO_4 (x ~ 0.5) nano-plates were harvested from the separate half-charged coin-cell (red solid line), that was interrupted at a capacity of 78 mAh g^{-1} (red solid cycle) during the first charge sequence.

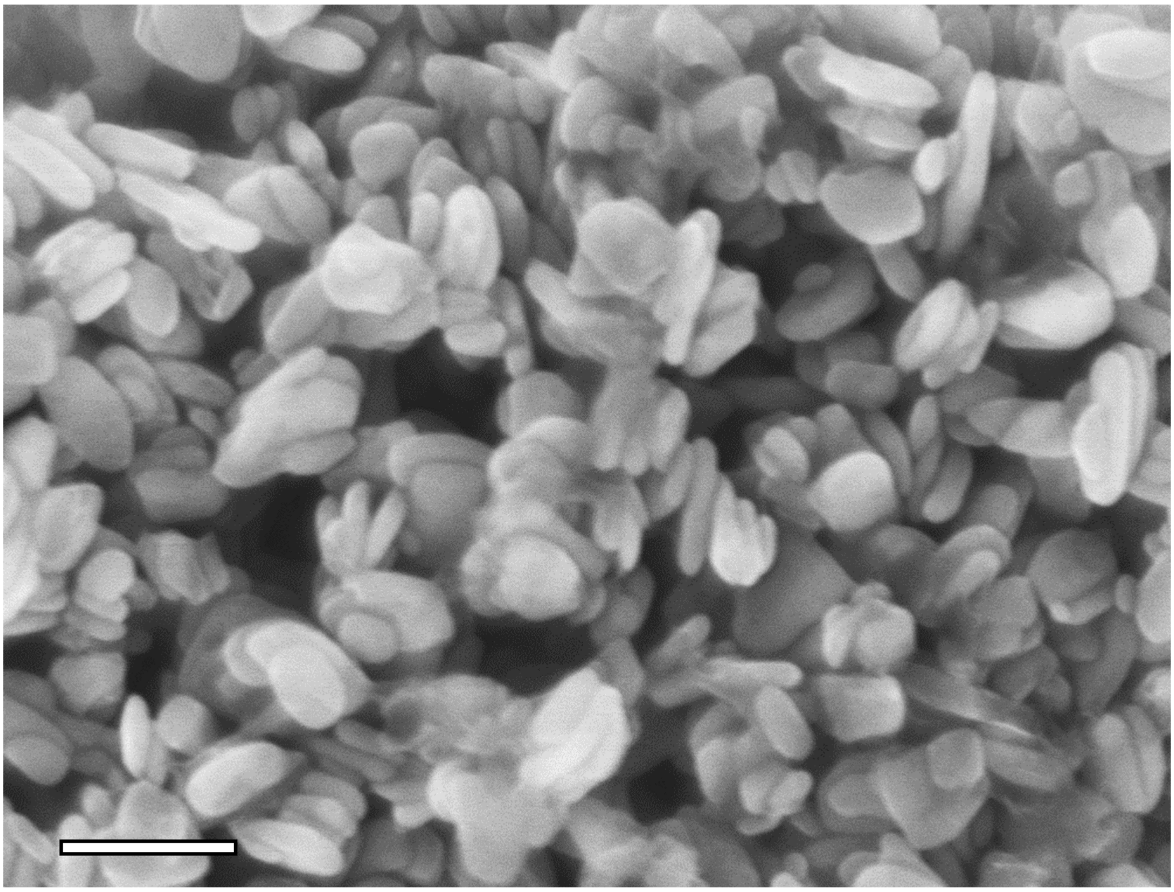

Supplementary Figure 2. Representative scanning electron microscopy (SEM) image of Li_xFePO_4. Scale bar, 200 nm.

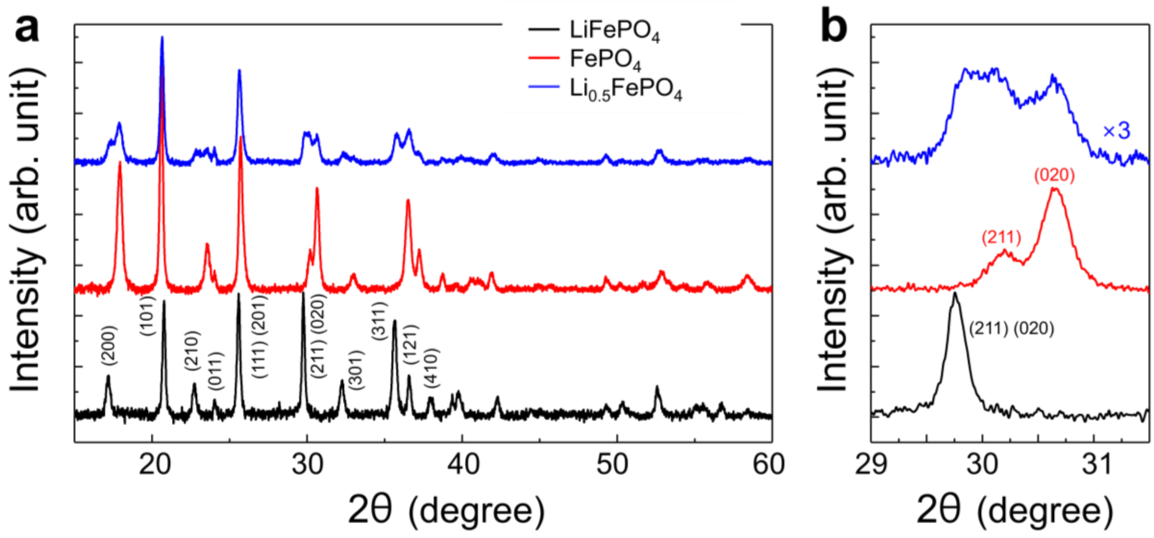

Supplementary Figure 3. X-ray diffraction analysis. a, X-ray diffraction patterns for pristine (black), partially (blue), and fully (red) delithiated $LiFePO_4$ nanoplates. Chemical delithiation to obtain $FePO_4$ was achieved by stirring $LiFePO_4$ for 1 hour in a bromine (0.05 *M*) acetonitrile solution. **b**, Detailed view for the region where the (020) reflections appear in $Li_{0.5}FePO_4$. The positions and relative intensities of peaks were consistent with a 50% ratio of $Li_{\alpha}FePO_4$ and $Li_{\beta}FePO_4$, where α and β were slightly smaller than 1 and very close to 0, respectively, according to comparisons with the extensive data in the literature[6].

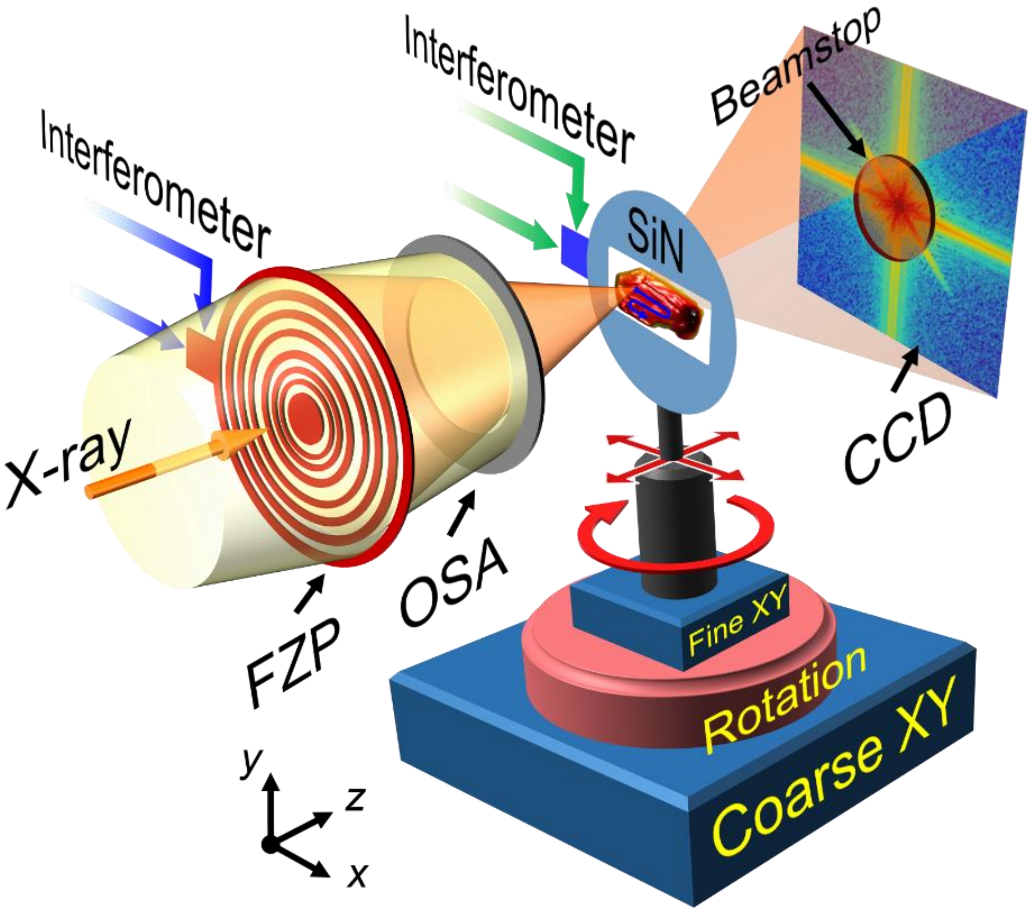

Supplementary Figure 4. Diagram of soft X-ray ptychographic microscopy. A monochromatic and coherent soft X-ray beam is focused onto the sample using a 100 nm outer-zone-width Fresnel zone plate (FZP). An order-sorting aperture (OSA) filters out higher-order diffraction. With mechanical raster motions of the FZP, the focused X-ray is scanned onto the sample to collect a series of diffraction patterns with 70 nm step to ensure overlap of the probed areas. Diffraction patterns are recorded on an X-ray charge-coupled device (CCD) positioned 80 mm downstream of the sample. To expand the dynamic range of the CCD, the central portions of the diffraction data are attenuated by the 5-μm-think Si_3N_4 attenuator. In order to record 2-dimensional images at various angular positions of the sample with respect to the X-ray beam, the sample is placed on a stage with high precision *x*, *y*, *z*, and θ (tomographic tilt angles) stage, which was stabilized by an optical interferometry (blue and green arrows).

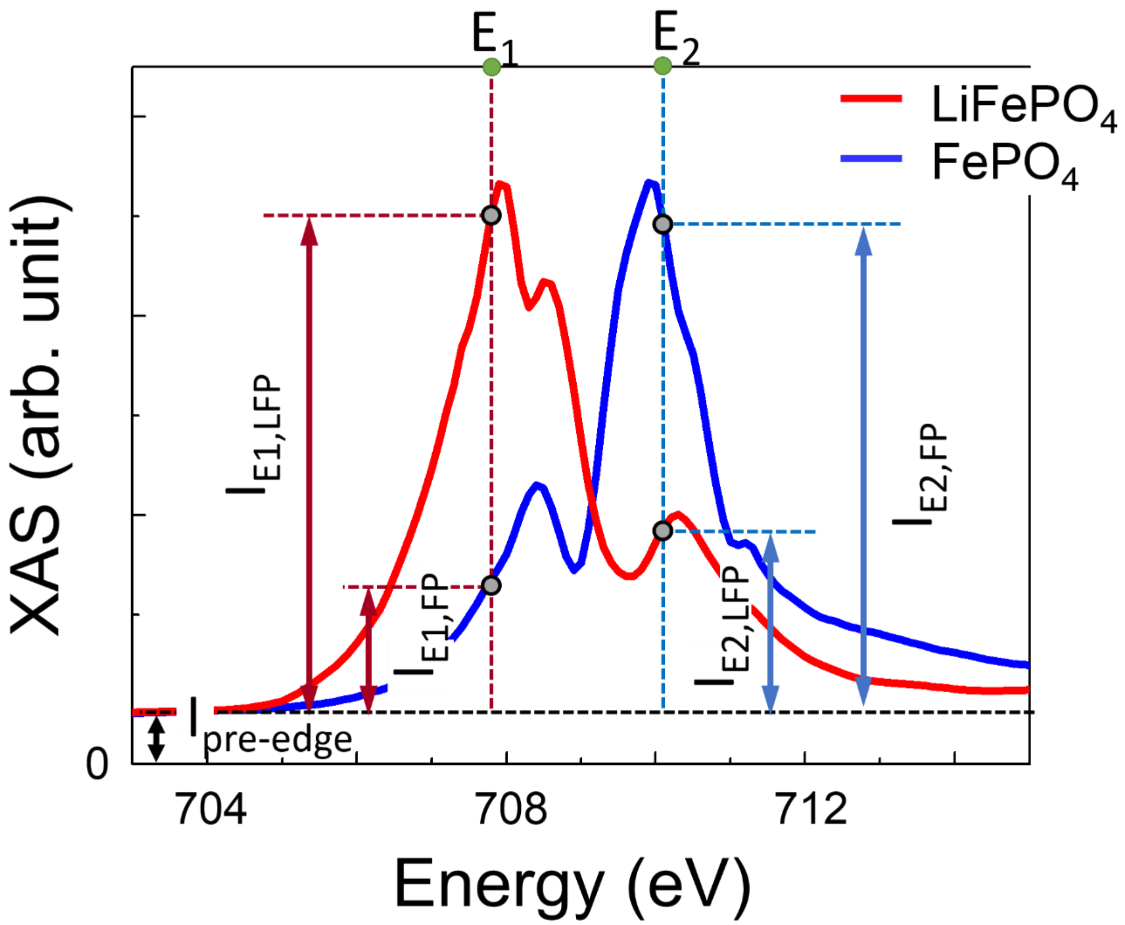

Supplementary Figure 5. Fe L_3 X-ray absorption reference spectra taken from pristine $LiFePO_4$ nanoplates and $FePO_4$ produced by chemical delithiation. To reduce the noise, over 15 spectra are averaged. Each spectrum was collected by linescan mode in conventional scanning X-ray microscopes (STXM) with energy step of 0.2 eV. The absorption at the specific energies (E_1 and E_2) are defined as $I_{E1,LFP}$, $I_{E1,FP}$, $I_{E2,LFP}$, and $I_{E2,FP}$. The pure mass thickness term in the reference spectra is defined as $I_{pre\text{-}edge}$.

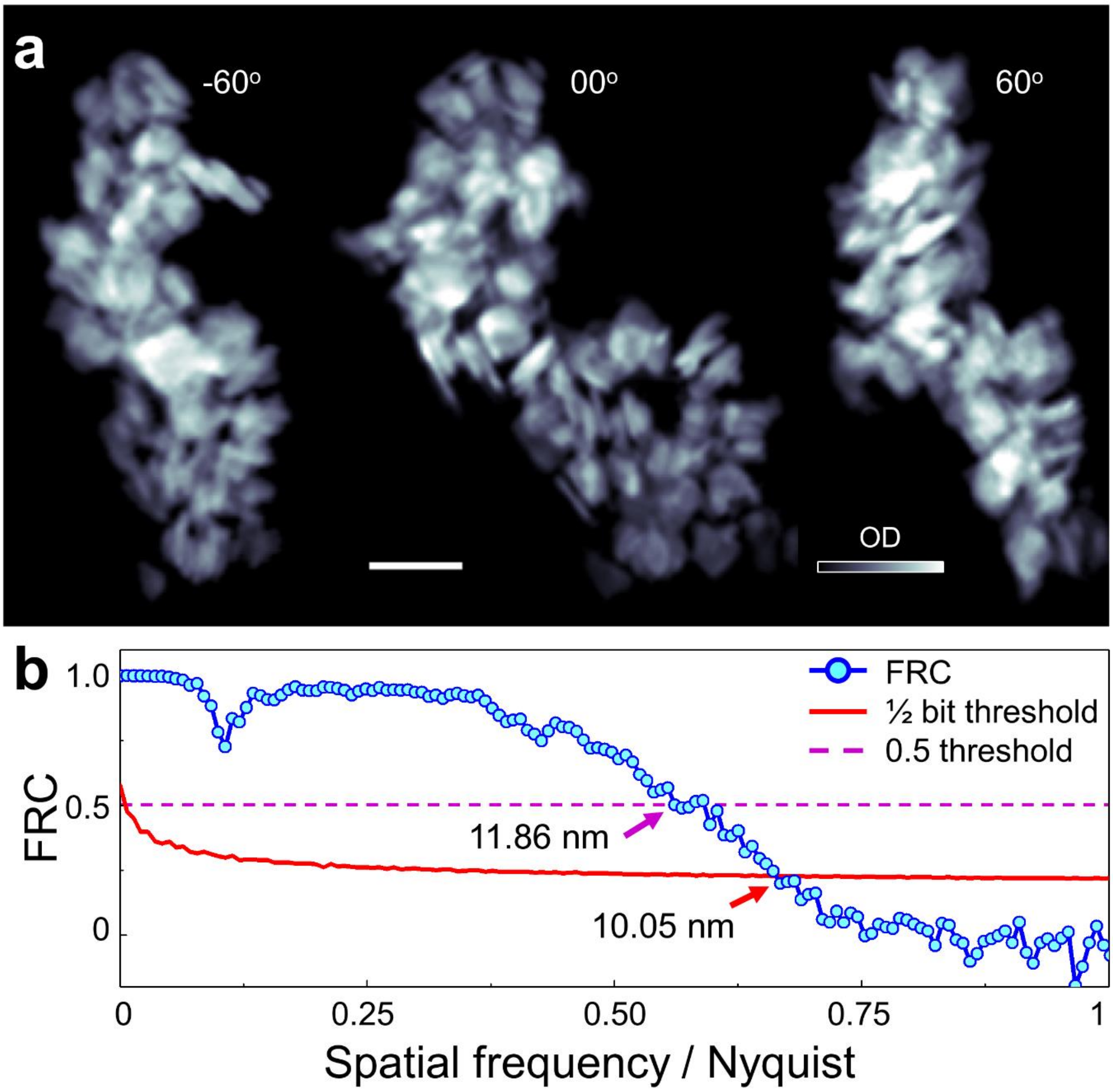

Supplementary Figure 6. Two dimensional spatial resolution. **a**, Phychographic reconstructions of two-dimensional (2D) optical density (OD) at specific tilt angles (*θ*) as noted. The size of reconstructed pixels is 6.7 × 6.7 nm^2. Scale bar, 200nm. **b**, Resolution estimation of the image at 0° in (**a**) by Fourier ring correlation (FRC, blue solid line with scatter) with 1/2-bit (red solid line) and 0.5 (magenta dashed-line) threshold criteria.

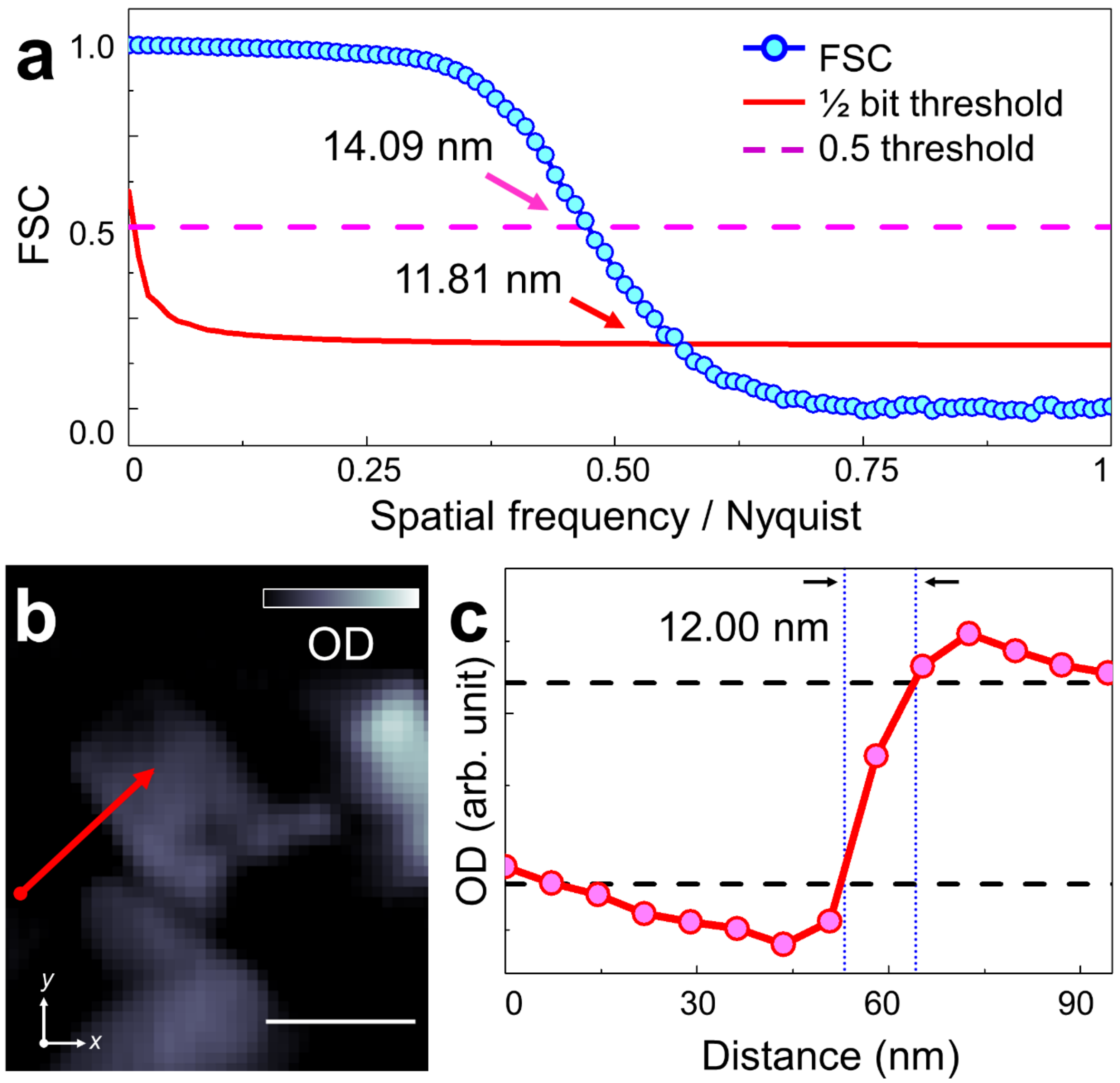

Supplementary Figure 7. Three dimensional spatial resolution at 710 eV. a, Resolution of the three-dimensional (3D) optical density (OD) volume at 710.2 eV in Fig. 1 by Fourier shell correlation (FSC, blue solid line with scatter) with 1/2-bit (red solid line) and 0.5 (magenta dashed-line) threshold criteria. **b**, Representative cross-section of the tomogram at 710.2 eV along the highest resolution plane (*xy*). The positions of the slices are marked as red arrows in Fig. 1a. Scale bar, 100 nm. **c**, Line profile indicated by the red arrow in **b**. Black-dashed lines are guides for 10-90% resolution criteria.

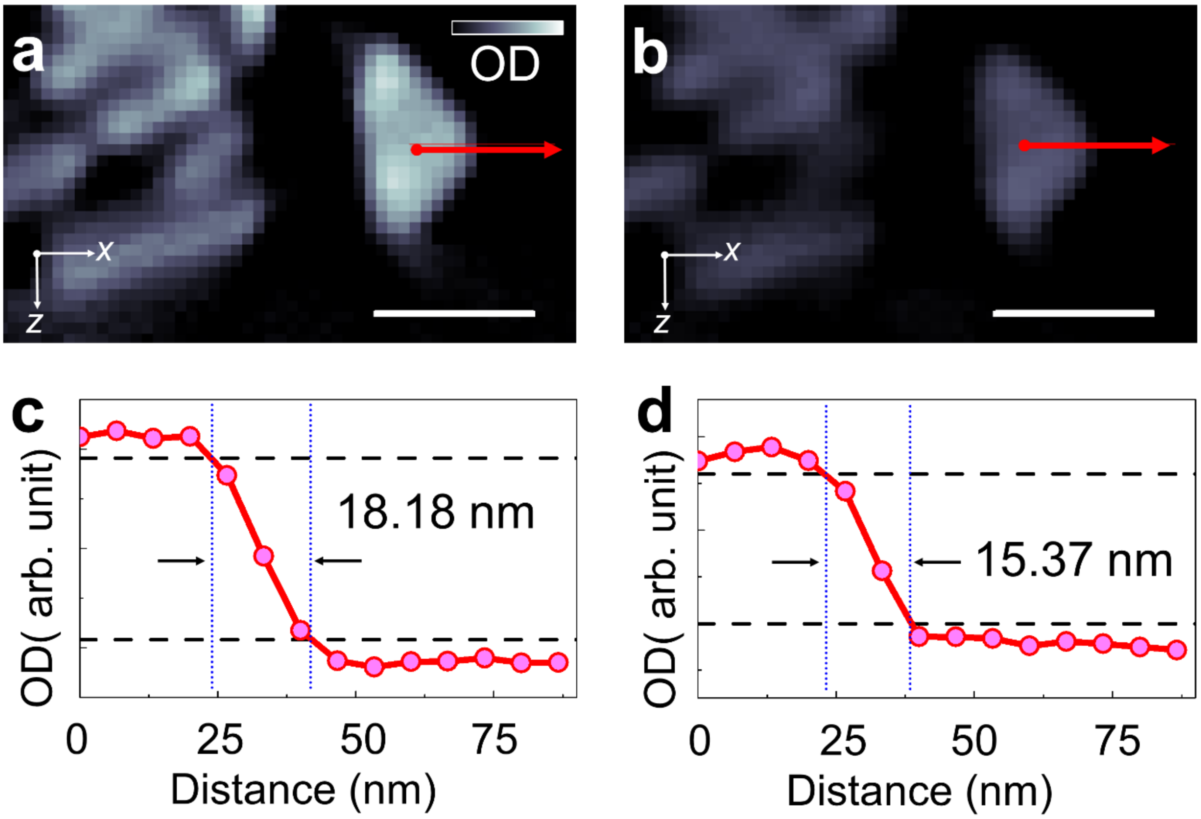

Supplementary Figure 8. Resolution along low-resolution planes. Representative cross-section of the tomogram at 708.2 (**a**) and 710.2 eV (**b**) along the low-resolution plane (*xz* plane). The positions of the slices are marked as blue arrows in Fig. 1a. Scale bars, 100 nm. **c, d,** Line profiles indicated by the red arrow in (**a**) and (**b**), respectively. Black-dashed lines are guides for 10-90% resolution criteria.

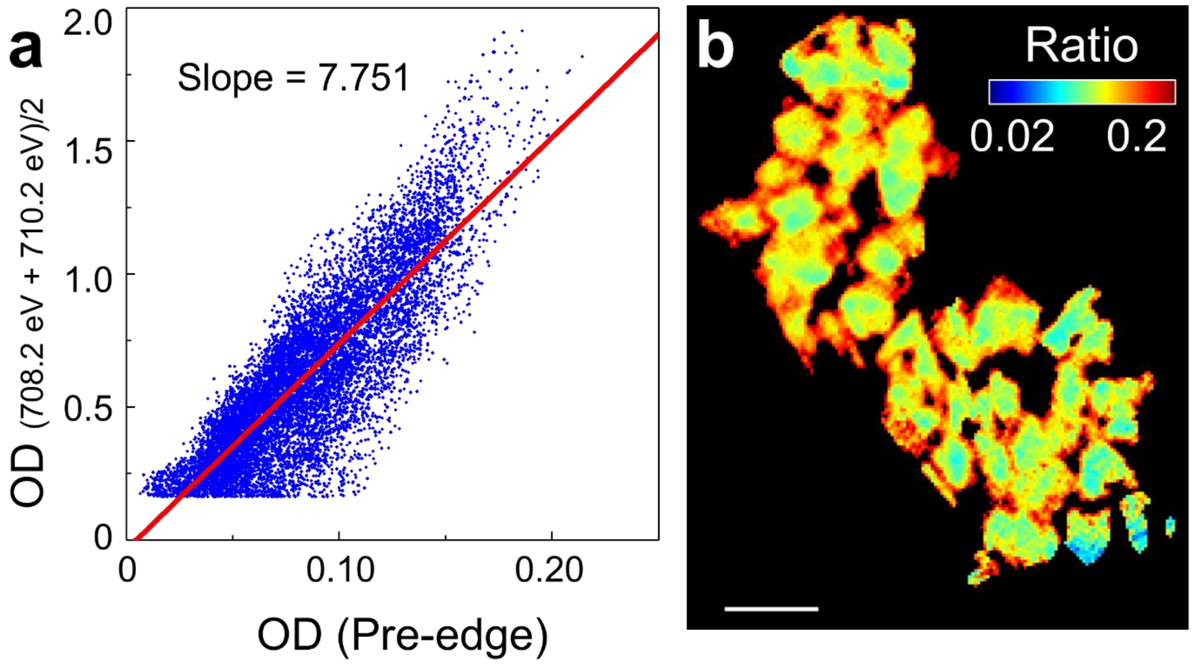

Supplementary Figure 9. Optical density ratio. a, Correlative distribution plots between the averaged optical density (OD) at two absorption peaks (708.2 and 710.2 eV) and the averaged OD at pre-edge region. To reduce noise in low contrast images at pre-edge region, three OD images (at 700.0, 702.7, and 705.2 eV) are averaged. The linear relation of correlative OD distribution is defined as a red sold line. **b**, OD ratio map between the averaged OD at two absorption peaks and the averaged OD at pre-edge region. Scale bar, 200 nm.

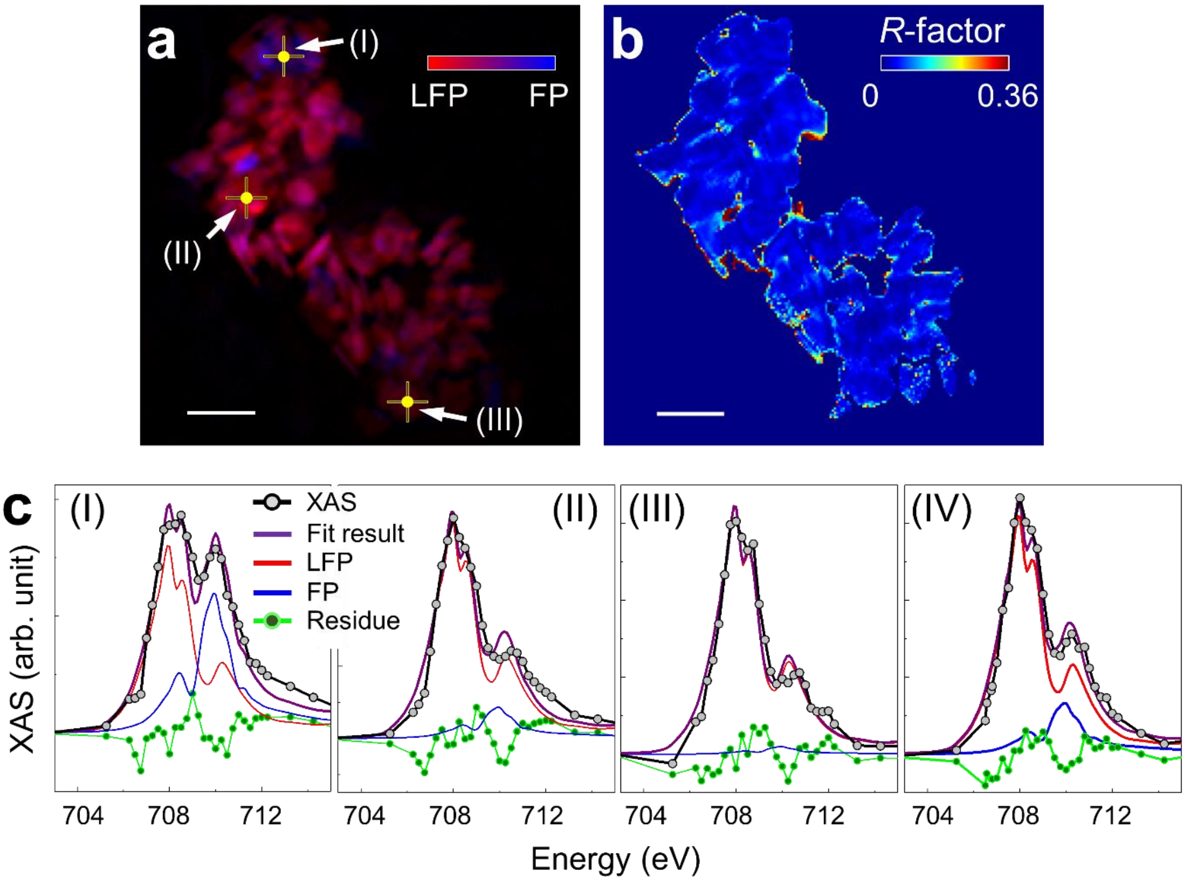

Supplementary Figure 10. Two dimensional chemical phase map. a, Two dimensional (2D) chemical phase maps obtained by linear combination (LC) fits of XAS data at each pixel. The presence of the $Li_{\alpha}FePO_4$ (LFP) and $Li_{\beta}FePO_4$ (FP) were assigned colors red and blue, respectively. The brightness indicates relative sample thickness along X-ray path. **b**, *R*-factor maps depicting the statistical quality of the single pixel fits in (**a**). Scale bars, 200 nm. **c**, Selected XAS spectra extracted from the marked positions in (**a**), and results of LC fits with standard spectra from $LiFePO_4$ (LFP) and $FePO_4$ (FP). For comparison purposes, XAS extracted from entire area of the object and its LC fit are shown in (IV). The residue is defined by the difference between XAS data and fit results.

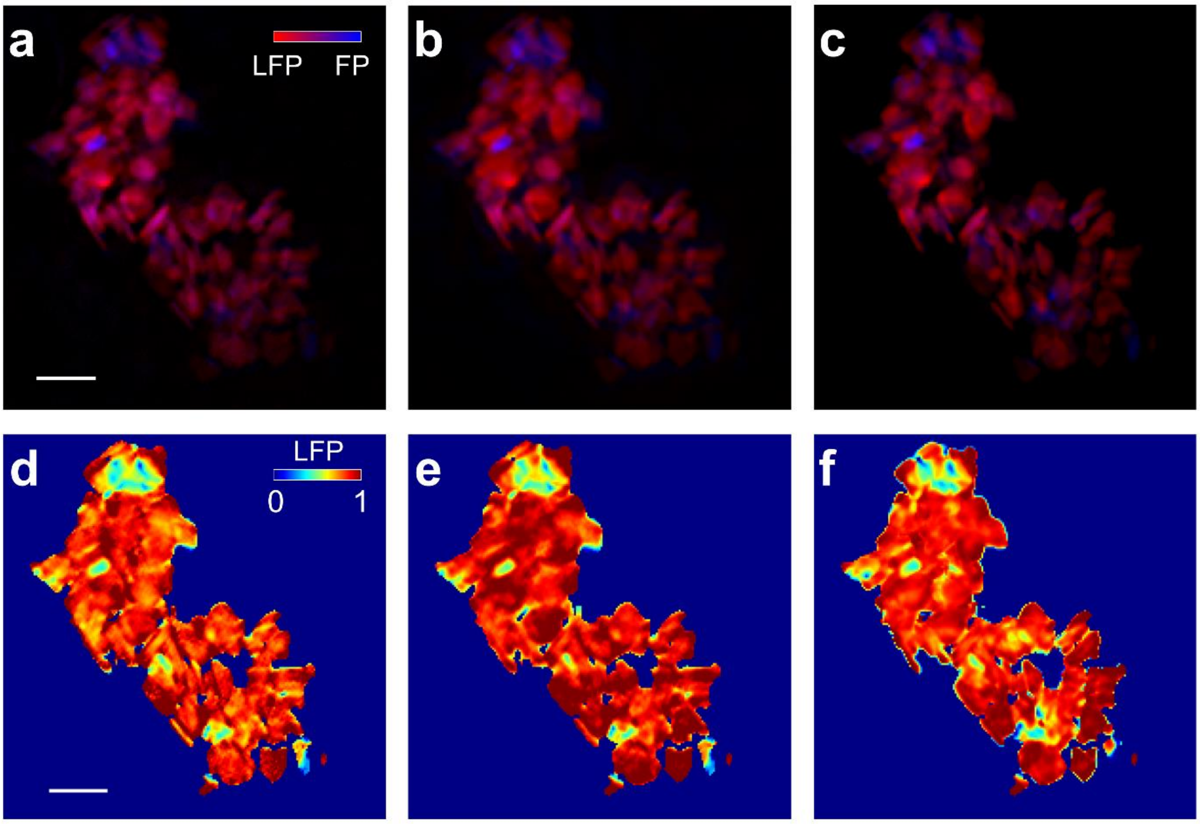

Supplementary Figure 11. Two dimensional chemical phase map. Two dimensional (2D) chemical phase maps from different methods; (**a**) linear combination (LC) fits of full XAS data; (**b**) the quantitative analysis with two optical density (OD) images at 708.2 and 710.2 eV; (**c**) the quantitative analysis with two OD images, which are 2D projections of three dimensional (3D) volume at 708.2 and 710.2 eV. The presence of the $Li_{\alpha}FePO_4$ (LFP) and $Li_{\beta}FePO_4$ (FP) were assigned colors red and blue, respectively. The brightness indicates relative sample thickness along X-ray path. **d**, **e**, **f**, heat map of chemical distributions, corresponding to the chemical maps in (**a**), (**b**), and (**c**), respectively. Here, the color contrast scales with the ratio of LFP contents in each pixel. Scale bars, 200 nm.

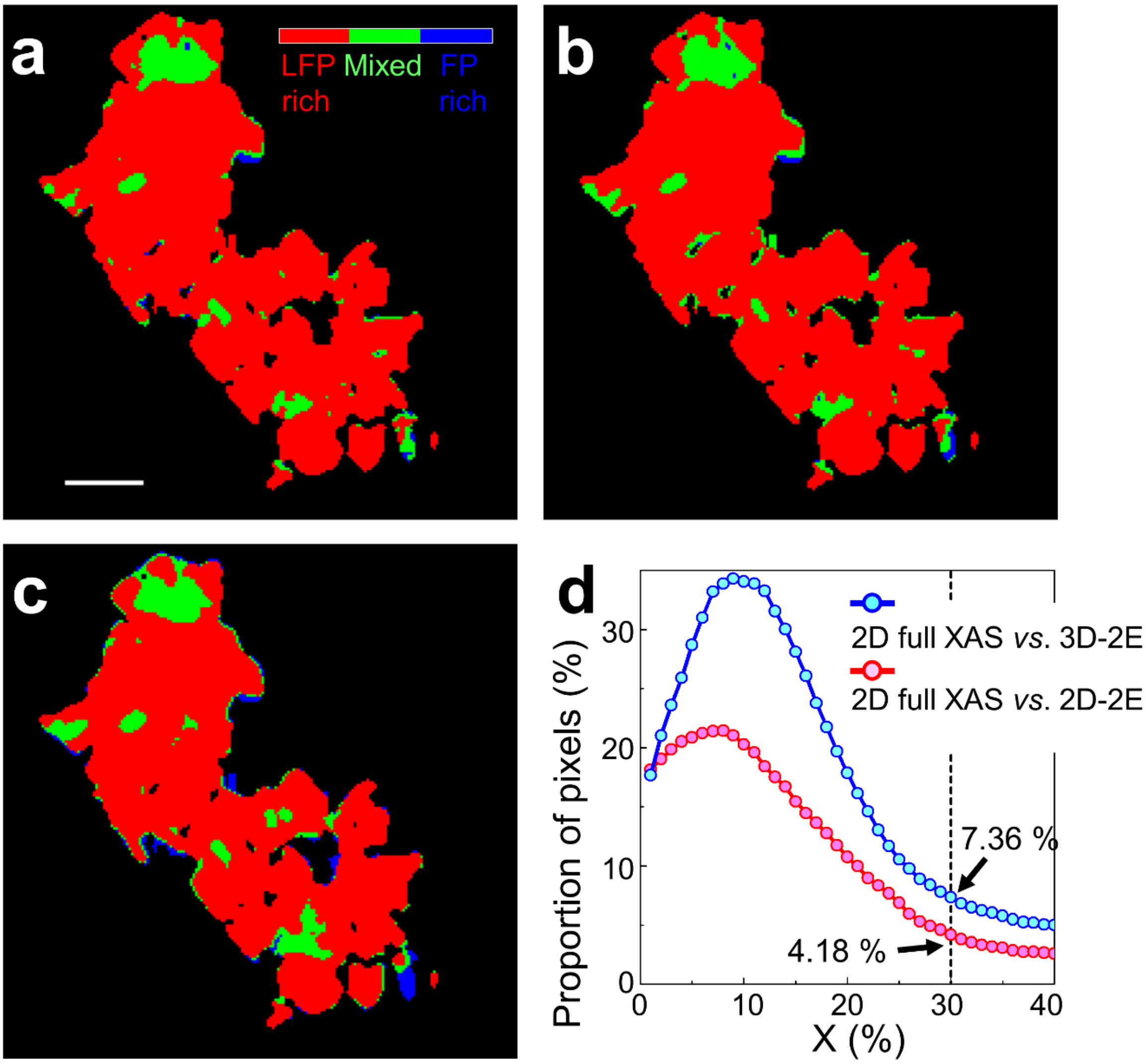

Supplementary Figure 12. Segmentations of chemical phases. Segmentation of two dimensional (2D) chemical phases with 30% threshold. The red, green, and blue areas indicate LFP-rich (>70% $Li_{\alpha}FePO_4$, $\alpha \geq 0.9$), FP-rich (>70% $Li_{\beta}FePO_4$, $\beta < 0.1$), and Mixed (30–70% $Li_{\alpha}FePO_4$, the rest being $Li_{\beta}FePO_4$) domains, respectively. Each 2D chemical phase map was obtained by different methods; (**a**) linear combination (LC) fits of full XAS data (indexed as 2D-full); (**b**) the quantitative analysis with two optical density (OD) images at 708.2 and 710.2 eV (indexed as 2D–2E); (**c**) the quantitative analysis with two OD images, which are 2D projections of three dimensional (3D) volumes at 708.2 and 710.2 eV (indexed as 3D–2E). **d**, Percentage of bad pixels which have mismatched chemical segments between (**a**) and (**b**) (red solid line with scatter) or (**a**) and (**c**) (blue solid line with scatter) according to the segmentation threshold (X). Scale bar, 200 nm.

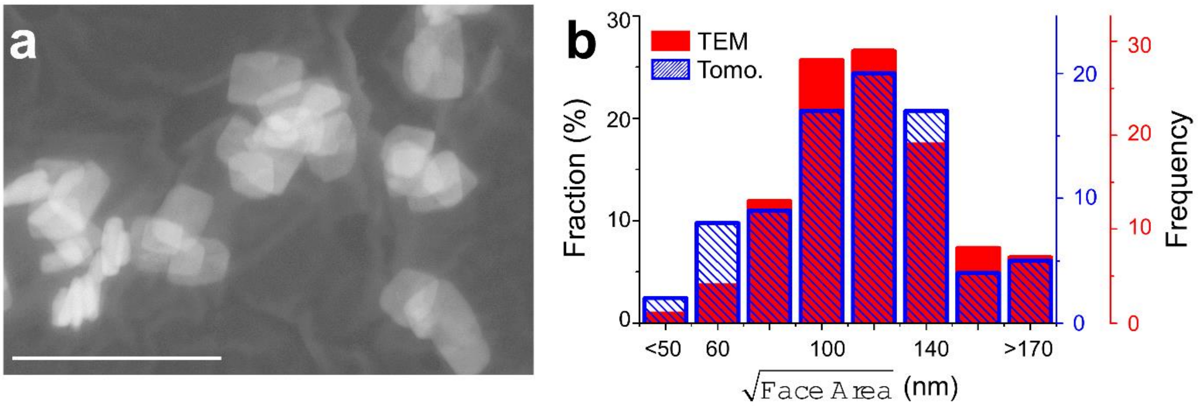

Supplementary Figure 13. Particle size distributio. a, Representative dark-field transmission electron microscopy (DF-TEM) image of pristine $LiFePO_4$. Scale bar, 500 nm. **b**, Histogram plot of square root of the facet areas. The total number of observed particles are 109 and 83 from the multiple TEM images (red solid bar) and the tomogram (blue hatched bar), respectively.

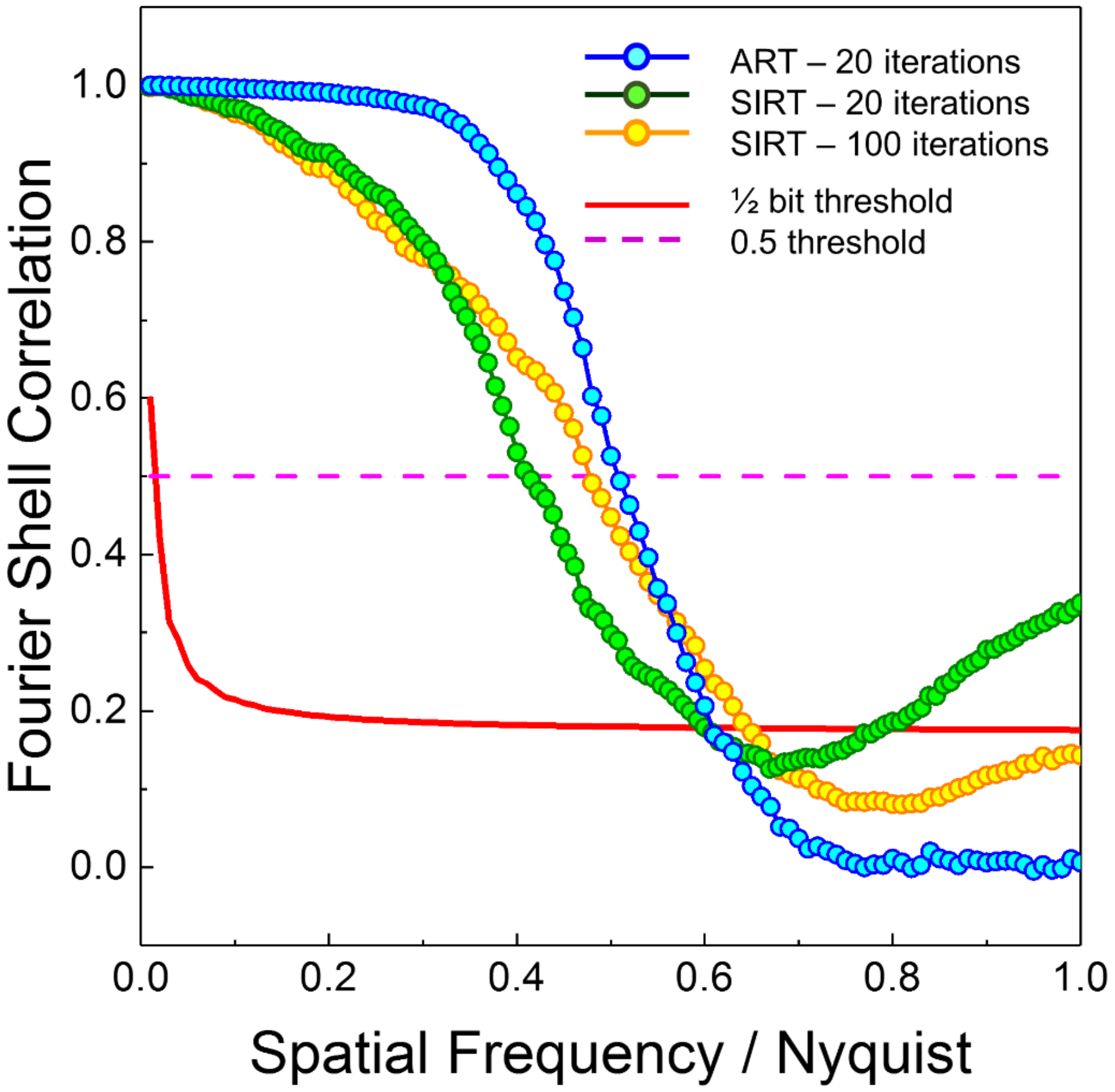

Supplementary Figure 14. Fourier shell correlation of the 3D volumes at 708.2 eV reconstructed by different reconstruction algorithms. The resolution estimations with 1/2-bit (red solid line) threshold criteria are 10.93 nm (ART – 20 iterations, blue solid line with scatter), 11.11 nm (SIRT – 20 iterations, green solid line with scatter), and 10.42 nm (SIRT – 100 iterations, orange solid line with scatter).

Supplementary Methods

Fourier ring correlation (FRC) For the FRC in Supplementary Fig. 6, the diffraction data at 0° were divided into two separate subsets by selecting every other measurement, which were then reconstructed as independent OD images of the same object[1]. The actual resolution should be somewhat higher as the FRC reduces the signal-to-noise ratio of the data by a factor of two.

Fourier shell correlation (FSC) In order to calculate the FSC, the 158 OD projections at each X-ray energy were divided into two separate subsets corresponding to even and odd tomographic tilt angles (θ), and then were reconstructed as an independent OD volume of the same object. The FSC of the lower contrast OD volume at 710.2 eV presents a 3D resolution of 12–14 nm (Supplementary Figs 7–8). Note that the actual resolution should be somewhat higher as the FRC reduces the signal-to-noise ratio of the data by a factor of two. These 3D resolutions are close to the theoretical resolution limit of 10 nm expected from the angular sampling of the sample diameter (i.e. approximately 0.5 μm in this case) based on the Crowther–DeRosier–Klug formula[2].

Quantitative 2D chemical phase map with X-ray absorption spectra A series OD images of the Li_xFePO_4 crystals across the Fe L_3 absorption edge were recorded by Soft X-ray ptychographic microscopy at 5.3.2.1 of the Advanced Light Source[3,4]. After ptychographic image reconstructions and pre-processing (e.g. image registration, background substations, reduction of sparkling noise, etc.), single pixel X-ray absorption spectra (XAS) were produced by integrating the OD across the energy. Reference spectra for pristine $LiFePO_4$ and chemically delithiated $FePO_4$ crystals were extracted from averaged linescans with

energy step of 0.2 eV (Supplementary Fig. 5). All single pixel spectra were fit by a linear combination (LC) of standard spectra (Supplementary Fig. 10) of $LiFePO_4$ and $FePO_4$. The quality of each LC fit can be checked with the *R*-factor map (Supplementary Fig. 10) defined as, $R = \sum(\text{data-fit})^2 / \sum(\text{data})^2$. 94.64% of pixels were fit with *R*-factors less than 0.15. Pixels showing poor signal-to-noise ratios were filtered out by a lower bound of the *R*-factor. Representative single pixel XAS and the corresponding fits are presented in Supplementary Fig. 10c.

Segmentation of chemical phases To reduce the error in the quantitative chemical map from two energy analysis, the chemical phases were segmented into three major components, such as LFP-rich, mixed, and FP-rich with the segmentation threshold (Supplementary Fig. 12). According to the segmentation threshold (X), each chemical component was defined as:

LFP-rich: > (100–X)% of $Li_\alpha FePO_4$ ($\alpha \geq 0.9$);

Mixed: (100–X) ~ X% $Li_\alpha FePO_4$, the rest being $Li_\beta FePO_4$ ($\beta < 0.1$);

FP-rich: > (100–X)% of $Li_\beta FePO_4$ ($\beta < 0.1$).

This segmentation, with $X = 30$, gives a clear view of the most reliable information and is in agreement with a similarly segmented 2D XAS map with a total error of 7.4%.

Segmentation of individual particles Dark and bright voxels in the 3D reconstructed OD volumes correspond to a region of weak absorption (e.g. porosity, carbon black, and polymer binder) and Li_xFePO_4 particles, respectively. To enhance the contrast, the ODs at 708.2 and 710.2 eV were averaged for representing the total iron content in the volume. The strong contrast in the OD volume was converted to level-based binary slices. The small features in the binarized slices were filled or removed before segmentation for avoiding over/under-

segmentation. We computed distance maps from binary images and created center positions for each segment from maxima regions of the distance map, then applied a 3D watershed algorithm[5]. Due to the weak contrast between proximate particles, the watershed algorithm caused inaccurate segmentation in some cases. To overcome these problems, we have introduced a criterion based on knowledge of the particle shape (i.e. $LiFePO_4$ has a plate-like shape in our case) and combined/separated to compensate for the inaccurate segmentation. Owing to unclear boundaries between some particles, a total of 27 particle bunches were not segmented, and the 83 individual particles were analyzed.

Supplementary References

1 Deng, J. *et al.* Simultaneous cryo X-ray ptychographic and fluorescence microscopy of green algae. *Proc. Natl. Acad. Sci. USA* **112**, 2314–2319 (2015).

2 Crowther, R. A., DeRosier, D. J. & Klug, A. The Reconstruction of a Three-Dimensional Structure from Projections and its Application to Electron Microscopy. *Proc. R. Soc. Lond. A* **317**, 319–340 (1970).

3 Shapiro, D. A. *et al.* Chemical composition mapping with nanometre resolution by soft X-ray microscopy. *Nat. Photonics* **8**, 765–769 (2014).

4 Yu, Y.-S. *et al.* Dependence on crystal size of the nanoscale chemical phase distribution and fracture in Li_xFePO_4. *Nano Lett.* **15**, 4282–4288 (2015).

5 Meyer, F. Topographic distance and watershed lines. *Signal Process.* **38**, 113–125 (1994).

6 Hess, M., Sasaki, T., Villevieille, C. & Novák, P. Combined *operando* X-ray diffraction–electrochemical impedance spectroscopy detecting solid solution reactions of $LiFePO_4$ in batteries. *Nat. Commun.* **6**, 8169 (2015).
