# Supplementary material for: Three-dimensional localization of nanoscale battery reactions using soft X-ray tomography
Source: Nat Commun. 2018 Mar 2;9:921. doi: 10.1038/s41467-018-03401-x (PMC5834601; doi:10.1038/s41467-018-03401-x)
Supplement: Supplementary file 3 — Description of Additional Supplementary Files [file 41467_2018_3401_MOESM3_ESM.pdf]

Description of Additional Supplementary File

File Name: Supplementary Movie 1

Description: Reconstructed 3-dimensional (3D) volumes of optical density (gray), chemical maps (red-blue), and its segmentation (red-green-blue). The size of reconstructed voxels is $6.7 \times 6.7 \times 6.7$ nm^3. The 3D edge contrast of the optical density volume is enhanced for clear visibility. The presence of the $Li_{\alpha}FePO_4$ (majority Fe^{2+}, LFP) and charged $Li_{\beta}FePO_4$ (majority Fe^{3+}, FP) were assigned colors red and blue, respectively (chemical map). The red, green, and blue areas indicate LFP-rich (>70% $Li_{\alpha}FePO_4$), FP-rich (>70% $Li_{\beta}FePO_4$), and Mixed (30–70% $Li_{\alpha}FePO_4$, the rest being $Li_{\beta}FePO_4$) domains, respectively (segmented chemical map).
